# Upregulated haptoglobin in classical monocytes serves as a diagnostic and immunological biomarker in myocardial infarction: a cross-sectional multi-omics study

**DOI:** 10.3389/fimmu.2025.1707912

**Published:** 2025-10-28

**Authors:** Hongchen Xu, Huibin Pan, Chanjuan Mo, Xueqi Guo, Longfei Ji, Danfei Shi, Binyu Wang, Guodong Li, Yong Li

**Affiliations:** ^1^ Emergency Intensive Care Unit, First Affiliated Hospital of Huzhou University, First People’s Hospital of Huzhou City, Huzhou, Zhejiang, China; ^2^ Department of Cardiovascular Center, First Affiliated Hospital of Huzhou University, First People’s Hospital of Huzhou City, Huzhou, Zhejiang, China; ^3^ Department of Clinical Laboratory, First Affiliated Hospital of Huzhou University, First People’s Hospital of Huzhou City, Huzhou, Zhejiang, China; ^4^ Department of Pathology, First Affiliated Hospital of Huzhou University, First People’s Hospital of Huzhou City, Huzhou, Zhejiang, China

**Keywords:** myocardial infarction, WGCNA, immune microenvironment, single-cell RNA sequencing, ferroptosis, exosomes

## Abstract

**Background:**

Myocardial infarction (MI) is one of the leading causes of death worldwide. Finding reliable diagnostic biomarkers and gaining a deeper understanding of their role in the immune microenvironment is of great significance for improving clinical prognosis.

**Method:**

This study integrated multiple datasets from GEO (GSE141512, GSE95368, GSE269269) and TCGA data, and used various bioinformatics methods such as weighted gene co-expression network analysis (WGCNA), immune cell infiltration analysis, and single-cell RNA sequencing analysis to screen key genes related to the occurrence and development of myocardial infarction. We initially validated the results using a proteomic dataset (GSE95368) and clinical samples analyzed by qPCR. Critically, the dysregulation and diagnostic value of Haptoglobin (HP) were further confirmed in multiple independent external cohorts (GSE66360, and others.), solidifying its reliability as a biomarker.

**Result:**

The study found that Haptoglobin (HP) is a key gene significantly upregulated in myocardial infarction, and it exhibits high diagnostic value (AUC=0.833) in the proteomic dataset (GSE95368). Single-cell sequencing analysis showed that HP is significantly highly expressed in classical monocyte of MI patients, and this finding was validated by qPCR experiments in clinically collected classical monocytes samples (p<0.05). Functional enrichment analysis implicated HP in immune responses and ferroptosis.

**Conclusion:**

The HP gene is a potential diagnostic biomarker for myocardial infarction, and its specific high expression in classical monocytes implies a potential role in the pathological process of myocardial infarction by regulating the immune microenvironment. This study provides a new research direction for the diagnosis and immune-targeted therapy of myocardial infarction, and has important clinical translational value.

## 1 Introduction

Myocardial infarction (MI) represents a critical global health burden with complex pathophysiology involving ischemia-driven cardiomyocyte death, inflammatory activation, and tissue remodeling (1). Despite established diagnostic markers like cardiac troponins (2), which are elevated after myocardial injury and serve as the gold standard for post-event diagnosis, a pressing need exists to identify novel molecular drivers that can aid in early risk stratification or pre-MI detection.

The advent of high-throughput genomics has enabled systems biology approaches to dissect the complexity of MI (3). Weighted gene co-expression network analysis (WGCNA) has emerged as a powerful tool to move beyond single-gene discoveries and identify functionally coherent gene modules associated with disease traits (4). Concurrently, two rapidly evolving biological concepts—ferroptosis (an iron-dependent form of regulated cell death) (5) and exosomes (6)-mediated intercellular communication—have been implicated in MI progression, yet their interplay and key regulators are poorly defined. Most previous studies have investigated these aspects in isolation, and a knowledge gap persists in the integrative analysis that converges co-expression networks with these specific phenotypic hallmarks of MI.

Furthermore, while bulk transcriptomic analyses provide valuable insights, they mask cellular heterogeneity. The application of single-cell RNA sequencing (scRNA-seq) in MI is now revealing the intricate contributions of specific immune cell subsets to the post-infarct inflammatory response (7). However, the expression and function of key regulators within this cellular ecosystem, as well as their trajectories over pseudo-time (a computational inference of temporal progression), remain largely unexplored.

**Figure 1:**
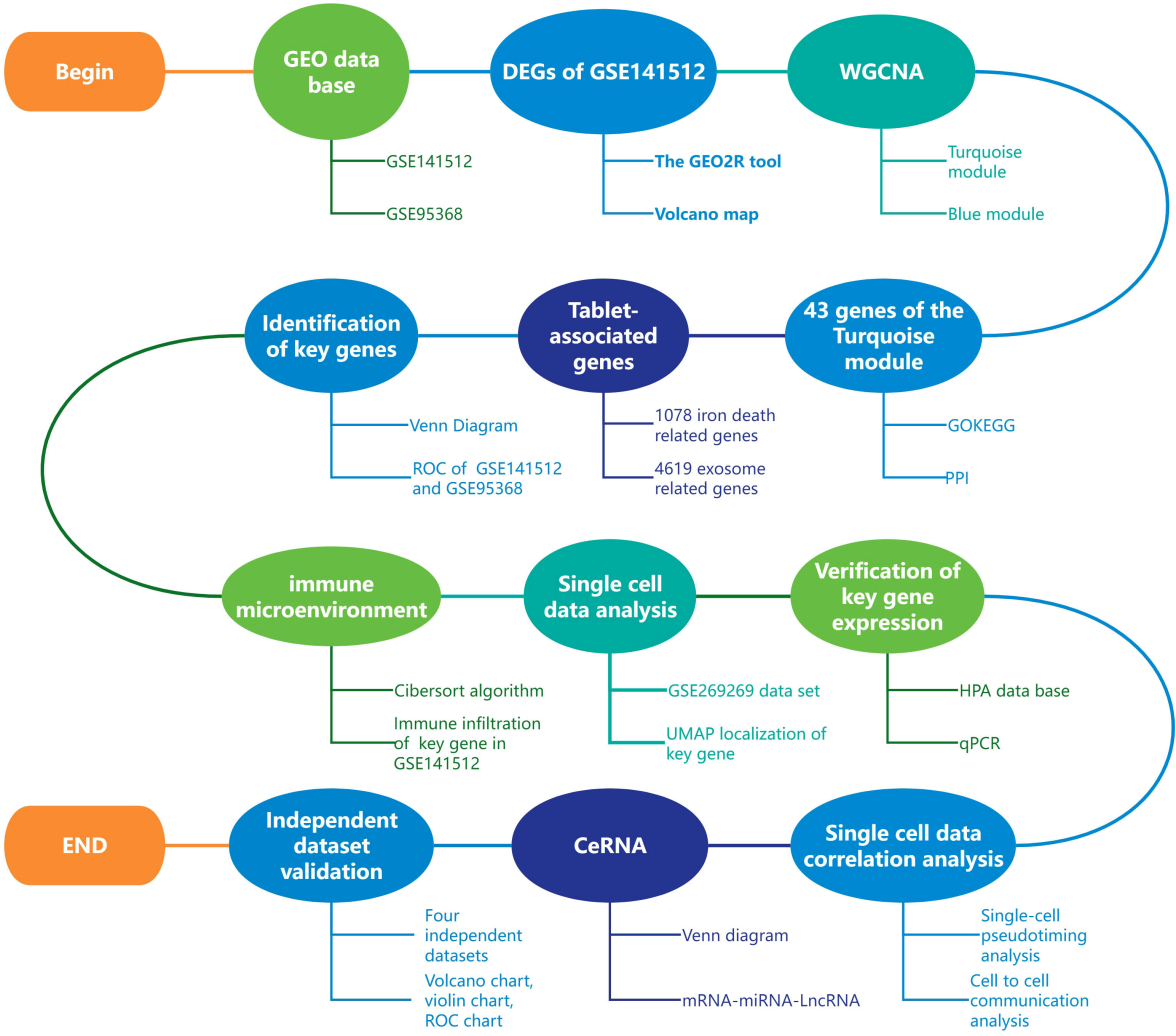
Article analysis flow chart.

Therefore, this study aims to identify and validate key immune-related genes in MI through an integrated multi-omics approach, with a focus on HP as a potential diagnostic biomarker and its role in the immune microenvironment and ferroptosis regulation. **Figure 1** shows the entire research process of the article.

## 2 Materials and methods

### 2.1 Data source

We used the GEO database (8) and three datasets were selected: GSE141512, GSE95368, and GSE269269. The details of these datasets are as follows:

GSE141512 (9): Platform number GPL17586 (HTA-2_0) Affymetrix Human Transcriptome Array 2.0 [transcript (gene) version]; includes 6 myocardial infarction (MI) patients and 6 healthy controls.

GSE95368 (10): Platform number GPL23119, SOMAScan human proteomic assay (SOMAScan assay 1.3k); contains 48 serum samples, including 12 myocardial infarction (MI) patients and 6 healthy controls selected for analysis.

GSE269269 (11): Platform number: GPL24676 Illumina NovaSeq 6000 (Homo sapiens), including 10 AMI patients (5 with plaque rupture and 5 without plaque rupture).

To independently validate the expression and diagnostic value of the identified key gene, four additional datasets were retrieved from the GEO database:

GSE66360 (12): This dataset, based on the GPL570 [HG-U133_Plus_2] Affymetrix Human Genome U133 Plus 2.0 Array platform, comprises a total of 99 peripheral blood samples, including 49 patients with myocardial infarction and 50 healthy controls. It serves as the primary large-cohort validation set.

GSE48060 (13): This dataset also utilizes the GPL570 platform and contains 52 peripheral blood samples from patients with coronary artery disease and normal controls.

GSE60993 (14): This dataset, based on theGPL6884 Illumina HumanWG-6 v3.0 expression beadchip, includes RNA-seq data from 24 samples (17 MI patients and 7 healthy donors).

GSE220865 (15): This dataset, based on the GPL20301 Illumina HiSeq 4000 platform, includes RNA-seq data from 15 samples (8 AMI patients and 7 healthy donors).

A detailed breakdown of the samples used from each dataset is provided in **Supplementary Table 1**.

### 2.2 Screening for differentially expressed genes

We extracted data from the GEO database using the GEO2R tool (16) and analyzed and visualized the downloaded dataset GSE141512 using R language (version 4.2.1) and Limma and ggplot2 packages (version 3.3.6). The criteria set for screening differentially expressed genes (DEGs) are p-value<0.05 and | logFC | ≥ 0 (17). Similarly, we analyzed the differential expression of Haptoglobin (HP) in four independent validation datasets (GSE66360, GSE48060, GSE60993, and GSE220865) using the same GEO2R tool. Evaluate the consistency of HP dysregulation across all cohorts using the same significance criteria.

### 2.3 WGCNA analysis

We performed weighted gene co-expression network analysis (WGCNA) on all genes in the GSE141512 dataset using the WGCNA R package (version 4.2.1). To focus on genes with biologically meaningful variations, we filtered out genes with low expression variability (standard deviation ≤ 0.5) prior to WGCNA, which is a common practice to reduce noise and enhance the reliability of network construction. Data visualization is achieved through the ggplot2 package (18) in R language (version 3.3.6).

### 2.4 GO/KEGG analysis and PPI network construction of turquoise module

In order to explore the functional significance of genes in the turquoise module, we performed Gene Ontology (GO) annotation and Kyoto Encyclopedia of Genes and Genomes (KEGG) pathway analysis on 43 genes (19). GO annotation and KEGG pathway analysis were performed using the DAVID tool (version 6.7) (20) to identify gene functions and associated signal transduction pathways. In addition, a protein-protein interaction (PPI) network was established through the STRING database to investigate the interactions between these genes (21).

### 2.5 Identification and validation of key phenotypic genes

Through the Genecards database (22), we collected a total of 1078 genes related to ferroptosis (see **Supplementary Table 2**) and 4619 genes related to Exosomes (see **Supplementary Table 3**). Subsequently, these phenotype-related genes were intersected with the turquoise module genes, and a Venn diagram was generated to identify the key gene HP. In the dataset GSE141512, we validated the receiver operating characteristic (ROC) curve of HP using the R package pROC (version 1.18.0) (23). In addition, we extracted data from GSE95368 using the GEO2R tool and visualized the volcano map (16). Subsequently, we evaluated the diagnostic value of HP in the GSE95368 dataset through ROC analysis.

### 2.6 Analysis of immune cell infiltration

This study used the Cibersort algorithm (24) to perform immune cell infiltration analysis on the GSE141512 dataset. By conducting correlation analysis, significant associations between target genes and various types of immune cells can be determined (**Figure 1**). All data analysis and visualization were completed using R software (version 4.2.1). We obtained data on 22 types of immune cells based on the CIBERSORTx website and analyzed them using codon sequence information (25). The core algorithm using R language script is used to detect gene expression profiles in the feature matrix, with P-value<0.05 as the criterion for significant correlation.

### 2.7 Analysis of single-cell RNA sequencing data

This study involved a sample of 10 myocardial infarction patients from the GSE269269 dataset (11). We used droplet-based single-cell RNA sequencing technology to analyze the collected samples, and the cell suspension was processed using microfluidic chips to improve the efficiency of cell capture and RNA extraction (26). The data processing uses the Seurat package (version 5.1.0) (27), which includes filtering, normalization, and scaling of cells and genes. Next, UMAP analysis (27) was performed to divide the cells into 17 clusters and further classify them into 10 cell types, such as eosinophils and classical monocytes.

### 2.8 HPA database verification

The Human Protein Atlas (HPA) public database (28) provides a large amount of proteomic and transcriptomic data from pathological and normal human tissues through immunohistochemistry (IHC) and RNA sequencing analysis. In this study, we evaluated the expression levels and distribution of the HP gene in immune cells in myocardial infarction (MI) specimens, and the design of these results was based on the HPA database, Monaco database, and Schmiedel database (29).

### 2.9 Single cell pseudo time series and cell communication analysis

In the R (version 4.2.1) environment, we import single-cell data, perform single-cell pseudo temporal analysis using the Monocle (version 2.23.0) package, and perform cell communication analysis using the CellChat (version 1.6.1) package (30). The Monocle package uses the DDRTree method for pseudo temporal inference, calculating pseudo time values to obtain the trajectory and state of cells during development (31). The analysis results are presented through various visualization methods, including cell trajectory distribution maps, temporal changes in gene expression, and heat maps. In the built-in functions of the CellChat package, we identify overexpressed genes and intercellular communication relationships, construct intercellular communication networks, and calculate communication strength (32). Finally, use the visualization functions provided by the CellChat package to draw circular, heatmap, and bubble plots of the communication network to demonstrate the communication relationships between different cell types.

### 2.10 ceRNA analysis

This study conducted competitive endogenous RNA (ceRNA) analysis on HP. We retrieved the miRNAs corresponding to HP from three databases (Target Scan (33), miRDB (34), and miRwalk (35)) and calculated the intersection using Venn plots to obtain five common miRNAs. Subsequently, long non-coding RNAs (LncRNAs) corresponding to these miRNAs were searched in the ENCORI database (36). Among the five candidate miRNAs, only hsa-miR-1247-5p was predicted to have lncRNA interactions in the ENCORI database. Therefore, we constructed a ceRNA network using Cytoscape (37) with hsa-miR-1247-5p as the core.

### 2.11 qPCR

This study used fluorescence quantitative PCR (qPCR) to validate HP expression levels in peripheral blood mononuclear cells from myocardial infarction (MI) patients. Researchers recruited 5 MI patients who were admitted to Huzhou First People’s Hospital from January 1, 2024 to February 28, 2024, and 5 healthy individuals who underwent routine health examinations during the same period as the control group. The inclusion criteria were: (1) age ≤ 90 years; (2) no history of radiotherapy or chemotherapy; (3) no fever or infection within 3 months before blood collection; (4) no history of blood transfusion.

We used density gradient centrifugation to separate human peripheral blood mononuclear cells. Peripheral venous blood was collected from the myocardial infarction patient group and the healthy control group, stored in heparin anticoagulant tubes, and mixed with an equal volume of 1 × PBS. The separation solution (P8680, Solarbio) was added in advance to a 15 mL centrifuge tube, and the sample was slowly layered on top to maintain clear separation (38). After centrifugation at 800 × g for 20–30 minutes, clear layers were formed in the tube, with a white cloud like ring in the middle representing PBMCs. Suck the middle layer, add more than 5 times the volume of PBS for resuspension, and wash twice by centrifugation at 250 × g to remove impurities. Finally, resuspend the cells in PBS and count to ensure cell viability>95%. The obtained PBMCs can be used for subsequent RNA extraction or stored at -80°C.

QPCR tests were performed following the MIQE guidelines (39). Three genes were selected to validate the RNA-seq results. QPCR primers were designed using Primer3 software (http://bioinfo.ut.ee/primer3-0.4.0/) and synthesized by Sangon Biotechnology Co., Ltd. (Shanghai, China) (40). For cDNA synthesis, 1μg of total RNA was reverse-transcribed using the PrimeScript RT reagent kit (TakaraBio™Inc., SAN Jose, CA) according to the manufacturer’s protocol. Quantitative RT-PCR was performed on the CFX96 Real-Time PCR system (Bio-RAD Laboratories, Hercules, CA, USA) using TB Green Premixed Ex Taq II (Takara Bio Inc). The consumables used included eight PCR tubes from Axygen^®^ brand products (Corning Corporation, Corning, New York, USA) (39). Each quantification used a 25 μL reaction mixture containing 12.5 μL TB Green Premix Ex Taq II, 1 μL (10 μM) of each primer, 8.5 μL RNase-free water, and 2 μL of 1:5 diluted cDNA. PCR amplification conditions consisted of initial denaturation at 95°C for 30 s, followed by 40 cycles of denaturation at 95°C for 5 s and annealing at 60°C for 30 s. After cooling to 65°C for 5s, the melting curve at the end of each PCR was obtained by gradually increasing the temperature to 95°C (with an incremental rate of 0.5°C/s). All samples underwent identical amplification analysis, eliminating the need for successive calibration. The data obtained were analyzed using Bio-Rad CFX Manager software (version 3.0), which generates raw quantitative cycle (Cq) values for each reaction using the 2-ΔΔCT method (40). The order of using primer pairs is as follows:

HP: Forward primer 5 ‘- cccgaaaaagacaccga-3’, reverse primer 5 ‘- gatcccgcgcataccagg-3’.

The research protocol has been approved by the Medical Research and Clinical Trial Ethics Committee of Huzhou First People’s Hospital (Approval No.: 2023KYLL013). All patients participating in this study provided informed consent.

### 2.12 Analysis of independent validation cohorts

The expression matrices for each validation dataset (GSE66360, GSE48060, GSE60993, GSE220865) were downloaded from the GEO database (8). The expression values of HP genes were extracted from each dataset. Evaluate the difference in HP expression between MI patients and healthy control group, with a significance threshold set at p<0.05. The results were visualized using the ggplot2 R package (version 3.3.6) (18). We evaluated the diagnostic performance of HP in each dataset by constructing receiver operating characteristic (ROC) curves and calculating the area under the curve (AUC) and its 95% confidence interval using the pROC R package (version 1.18.0) (23). In addition, this study used the Cibersort algorithm (24) to perform immune cell infiltration analysis on the GSE48060 and GSE66360 datasets. Through correlation analysis, significant associations between target genes and various types of immune cells can be determined. All data analysis and visualization were completed using R software (version 4.2.1) (**Figure 1**).

### 2.13 Statistical methods

All data analysis in this study was conducted using R language (version 4.2.1), and all statistical methods were analyzed using the corresponding R package. The P-value<0.05 was used as the standard for statistical significance, marked as * (p<0.05), * * (p<0.01), and * * * (p<0.001).

## 3 Result

### 3.1 WGCNA screening key module genes

**Figure 2:**
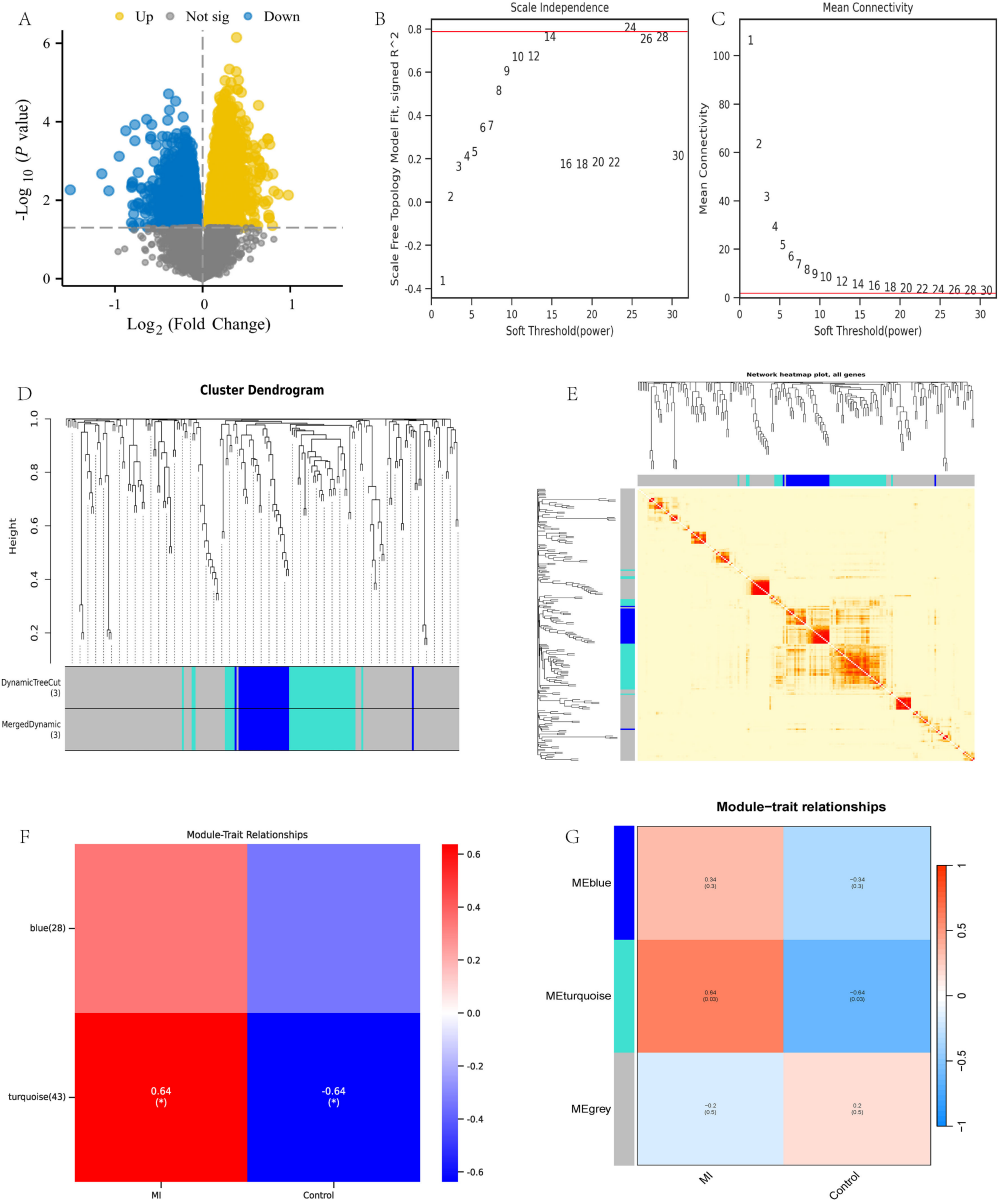
WGCNA analysis of dataset GSE141512. **(A)** GSE141512 volcano map; **(B, C)** Selection of WGCNA network construction parameters; **(D, E)** WGCNA module identification and analysis; **(F, G)** WGCNA module trait association analysis.

Using R language to analyze the dataset GSE141512, 27273 valid genes were obtained, including 5741 differentially expressed genes (DEGs). Specifically, there are 2500 upregulated genes and 3241 downregulated genes (**Figure 2A**). Afterwards, weighted gene co-expression network analysis (WGCNA) was performed on all valid genes (4). According to the power value of 24 used in this analysis (**Figures 2B, C**), WGCNA divides the genes into three modules: gray, turquoise, and blue (**Figures 2D, E**). The gray module includes gene sets that we could not classify into other modules and that lack reference significance. Based on the correlation between modules and traits, we selected the turquoise module for further analysis (**Figures 2F, G**).

### 3.2 GO/KEGG analysis and PPI network construction

**Figure 3:**
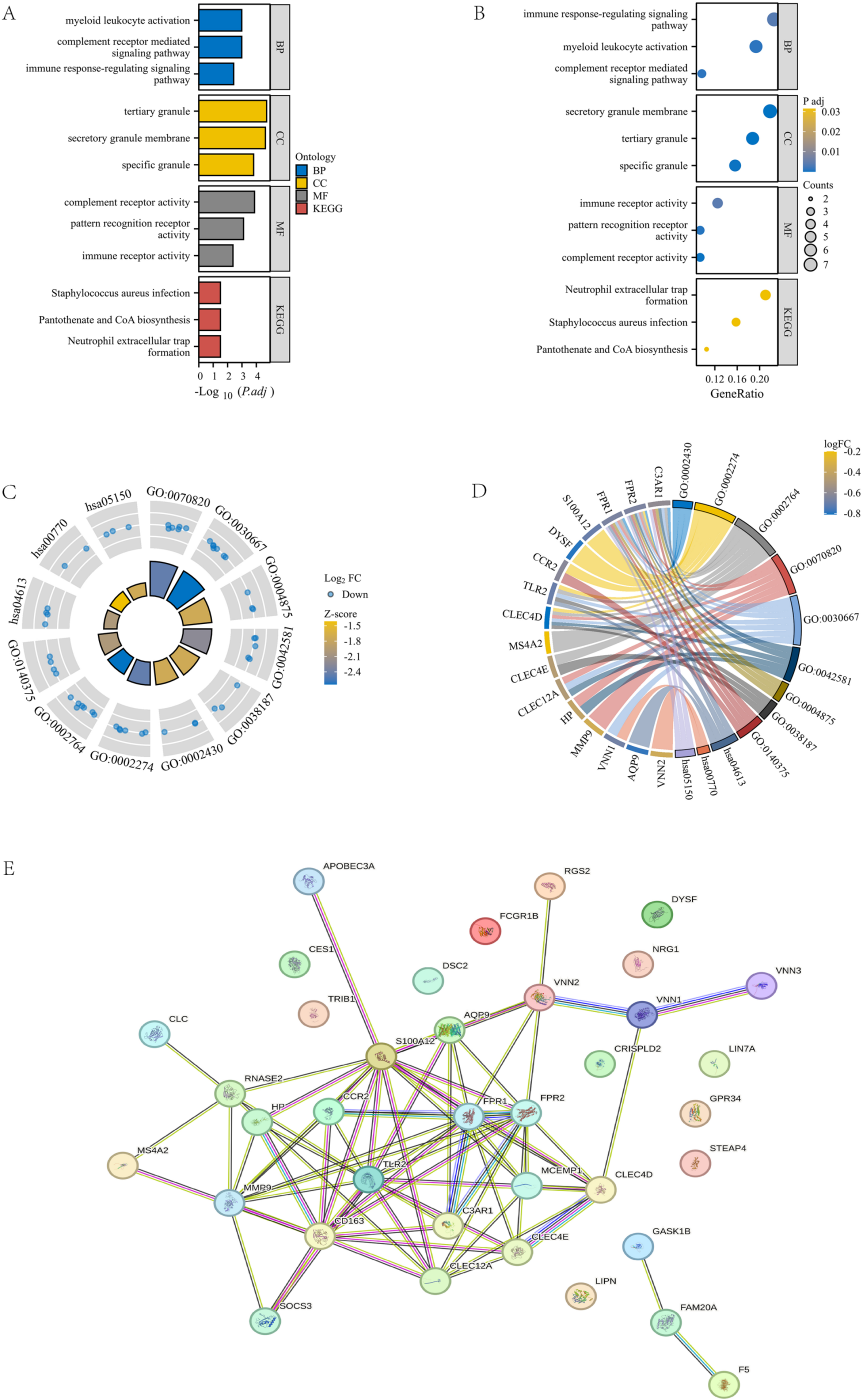
GOKEGG enrichment analysis and PPI construction of turquoise module. **(A, B)** Bar chart and bubble plot of GOKEGG enrichment analysis of turquoise module genes. **(C, D)** Circle plot and chord plot of GOKEGG enrichment analysis of turquoise module genes combined with logFC values. **(E)** PPI network diagram of turquoise module genes in STRING database.

To further analyze the gene functions in the turquoise module, we conducted Gene Ontology (GO) and KEGG pathway analyses on 43 genes and obtained the following results: Biological Process (BP): activation of myeloid leukocytes, complement receptor-mediated signaling pathways, and immune response regulatory signaling pathways; Cellular Component (CC): tertiary granules, secretory granule membrane, specific granules; Molecular Function (MF): Complement receptor activity, pattern recognition receptor activity, immune receptor activity; KEGG pathway: Staphylococcus aureus infection, pantothenic acid and coenzyme A (CoA) biosynthesis, neutrophil extracellular trap formation (**Figures 3A, B**). Next, we combined the logFC values of these genes to create separate circular and chord plots for observing the relationships between genes (**Figures 3C, D**). In addition, we searched for these 43 module genes in the STRING database and successfully established a protein-protein interaction (PPI) network containing 37 genes (**Figure 3E**). These findings reveal the important role of turquoise module genes in multiple functional pathways.

### 3.3 Identification and validation of genes related to exosomes and ferroptosis phenotype

**Figure 4:**
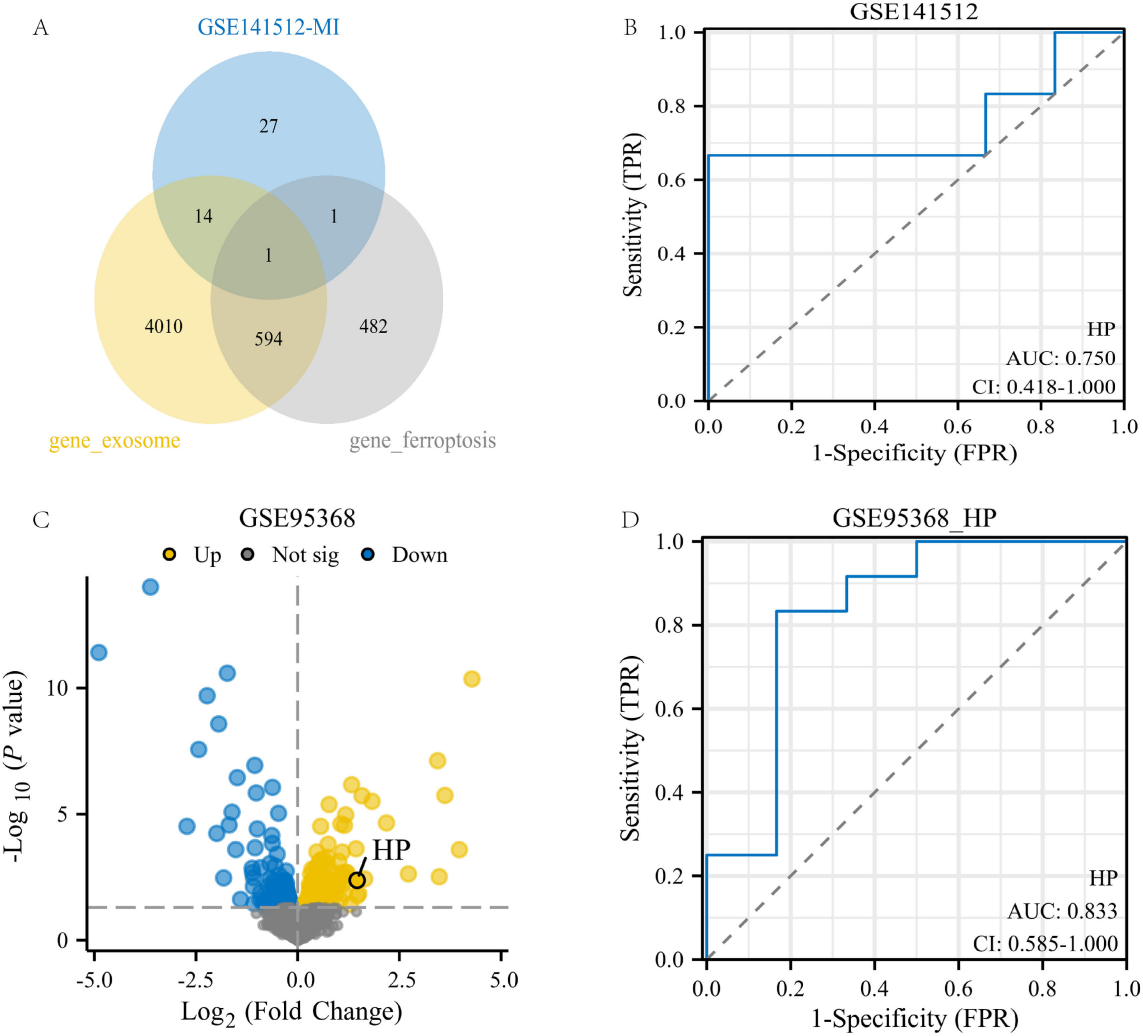
Identification and validation of genes related to exosomes and ferroptosis phenotype. **(A)** Intersection of the 43geturquoise module gene of MI with genes related to exosomes and ferroptosis phenotype; **(B)** ROC analysis of HP gene in GSE141512 dataset; **(C)** Volcanic map of GSE95368 dataset;**(D)** ROC analysis of the HP gene in the GSE95368 dataset.

This study conducted an intersection analysis between the turquoise module gene and genes related to ferroptosis and exosomes. Ultimately, a key gene was identified: HP (Haptoglobin gene, **Figure 4A**). This gene is located on human chromosome 1 and mainly encodes Haptoglobin protein, which is a plasma protein mainly synthesized in the liver. Its main function is to bind free hemoglobin to prevent it from freely existing in the blood and causing oxidative damage (41).

To validate the potential value of the HP gene in myocardial infarction (MI), we conducted validation analysis on two datasets, GSE141512 and GSE95368. The results showed that in the GSE141512 dataset, receiver operating characteristic (ROC) analysis of HP gene expression yielded an AUC of 0.75 (**Figure 4B**). In the GSE95368 dataset, HP was significantly upregulated in regions highlighted by volcano plots (p<0.05, log2FC≥0), with an AUC of 0.833 in ROC analysis (**Figures 4C, D**). These results indicate that the HP gene has high expression and good diagnostic value in MI, and may be a key gene promoting the occurrence and development of MI.

### 3.4 Immune infiltration analysis and validation

**Figure 5:**
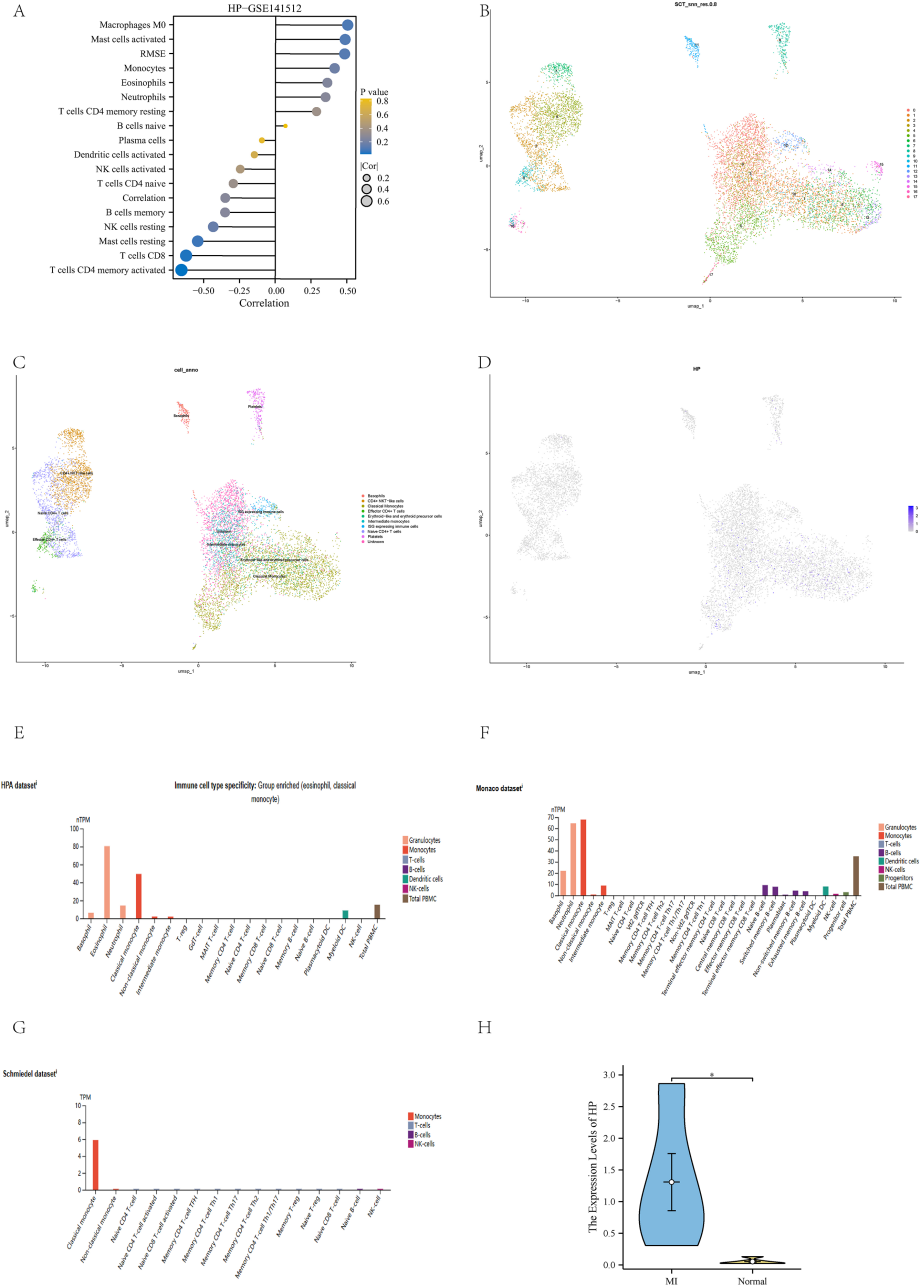
Immune infiltration analysis and validation. **(A)** Immune infiltration analysis of HP in GSE141512 dataset; **(B)** UMAP clustering of GSE269269 dataset; **(C)** Cell annotation of GSE269269 dataset; **(D)** The position of HP in the UMAP clustering map; **(E)** Immune cells with high expression of HP in HPA dataset; **(F)** Immune cells with high expression of HP in the Monaco dataset; **(G)** Immune cells with high expression of HP in Schmiedel dataset; **(H)** QPCR verification showed that HP was highly expressed in classical monocytes of MI compared to the control group.

We applied the Cibersort algorithm to analyze the GSE141512 dataset and found significant correlations between HP and various immune cells, including M0 macrophages, activated mast cells, RMSE, and classical monocytes (**Figure 5A**). Subsequently, this study rigorously screened single-cell RNA sequencing (scRNA seq) data from 10 patients with acute myocardial infarction (AMI) from the GSE269269 dataset. Through UMAP analysis (27), all cells were divided into 17 clusters (**Figure 5B**), which were further classified into 10 different cell types, including eosinophils, CD4+NKT like cells, classical monocytes, and effector CD4+T cells (**Figure 5C**). Among these cell types, HP is mainly highly expressed in classical monocytes (**Figure 5D**), which is consistent with the positive correlation between HP and classical monocytes in immune infiltration analysis. In addition, we found a graph of high expression of HP genes in immune cells, especially in classical monocytes, in the HPA database. This result not only involves the HPA dataset, but also includes the Monaco dataset and Schmiedel dataset (**Figures 5E–G**) (29). To verify the credibility of this discovery, we collected blood samples from MI patients who received treatment in our hospital and compared them with healthy individuals who underwent routine health checks during the same period as the control group. We extracted classical monocytes from whole blood and performed qPCR validation. The results showed that HP was significantly higher in classical monocytes of MI patients than in the control group (p<0.05) (**Figure 5H**). Therefore, these results fully demonstrate that HP significantly affects the immune microenvironment of MI through high expression in classical monocytes.

### 3.5 Time series analysis and cell communication analysis

**Figure 6:**
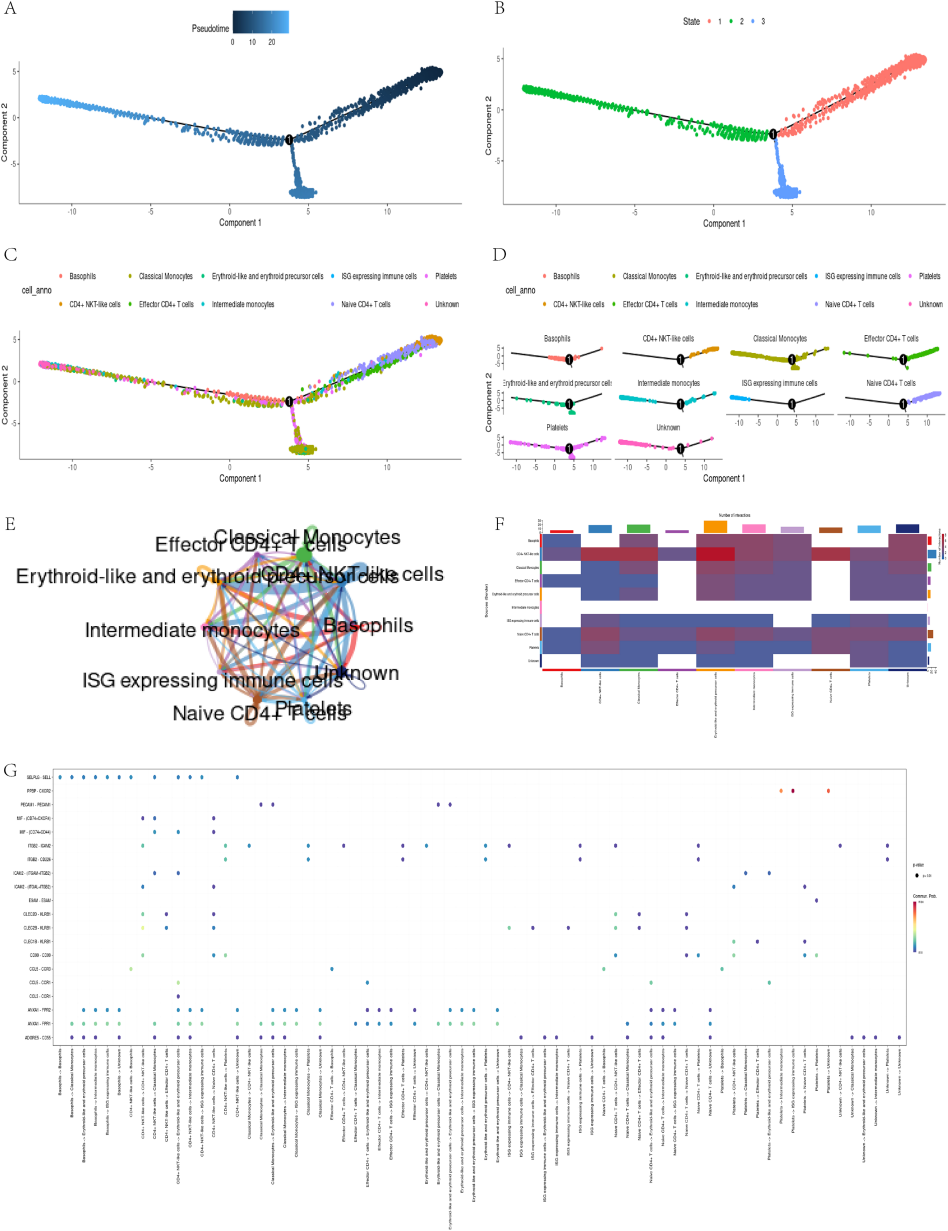
Pseudo-temporal trajectory and intercellular communication analysis. **(A, B)** Trajectory of cell states; **(C, D)** Distribution of classical monocytes over pseudo-time; **(E-G)**CellChat analysis reveals strong communication between classical monocytes and CD4+ T cells.

Through pseudo temporal analysis, we successfully obtained three differentiation stages (**Figures 6A, B**). The analysis shows that CD4+ T cells significantly increase during the early stages, reflecting their critical role in early immune responses. Classical monocytes peak in the middle and late stages, which may relate to remodeling of the myocardial infarction (MI) microenvironment, indicating their potential role in the subacute phase of MI (**Figures 6C, D**).

In addition, through the visualization tool of CellChat software (30), we have constructed various charts to display the communication relationships between different cell types. These visual charts reveal complex cellular interaction patterns, helping researchers better understand cellular communication mechanisms. The circular diagram illustrates the cellular communication network, with the thickness of the lines representing the strength of the connections (**Figure 6E**); The heatmap presents an overall overview of communication intensity, with color depth reflecting the degree of difference (**Figure 6F**); The bubble chart vividly illustrates the diversity of communication, with the size and color of bubbles representing interaction frequency and importance, respectively (**Figure 6G**). In summary, these charts depict communication relationships between cells and promote a deeper understanding of their interactions. They provide valuable data for research and drive innovation in the field of cell communication.

### 3.6 CeRNA analysis

**Figure 7:**
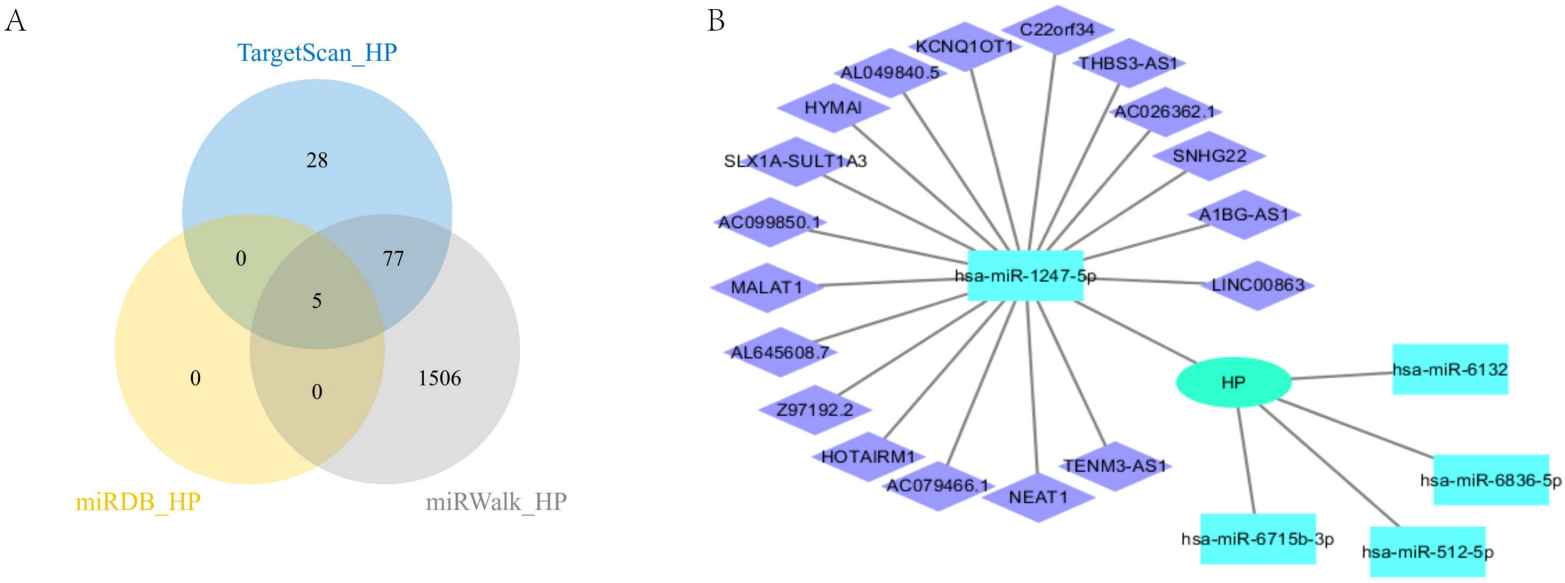
CeRNA analysis of HP. **(A)** HP’s miRNA intersection in three databases; **(B)** CeRNA analysis of HP.

In this study, we conducted a comprehensive analysis of the microRNA (miRNA) regulatory network associated with HP. **Figure 7A** clearly shows five overlapping miRNAs identified from three databases (Target Scan (33), miRDB (34), and miRwalk (35)), namely hsa-miR-512-5p, hsa-miR-1247-5p, hsa-miR-6715b-3p, hsa-miR-6836-5p, and hsa-miR-6132 (**Figure 7A**). We subsequently searched for the long non-coding RNAs (LncRNAs) corresponding to these miRNAs in the ENCORI database (36), and only obtained LncRNAs related to hsa-miR-1247-5p. We then constructed a protein-protein interaction (PPI) network using Cytoscape (37) (**Figure 7B**). This analysis provides important evidence for understanding the multi-level mechanisms of HP regulation and its impact on cellular processes. It highlights the complex interactions between miRNAs and HP. These findings lay the foundation for further exploration of miRNA functions in regulating gene expression under various physiological and pathological conditions.

### 3.7 Verification of HP in an independent dataset

**Figure 8:**
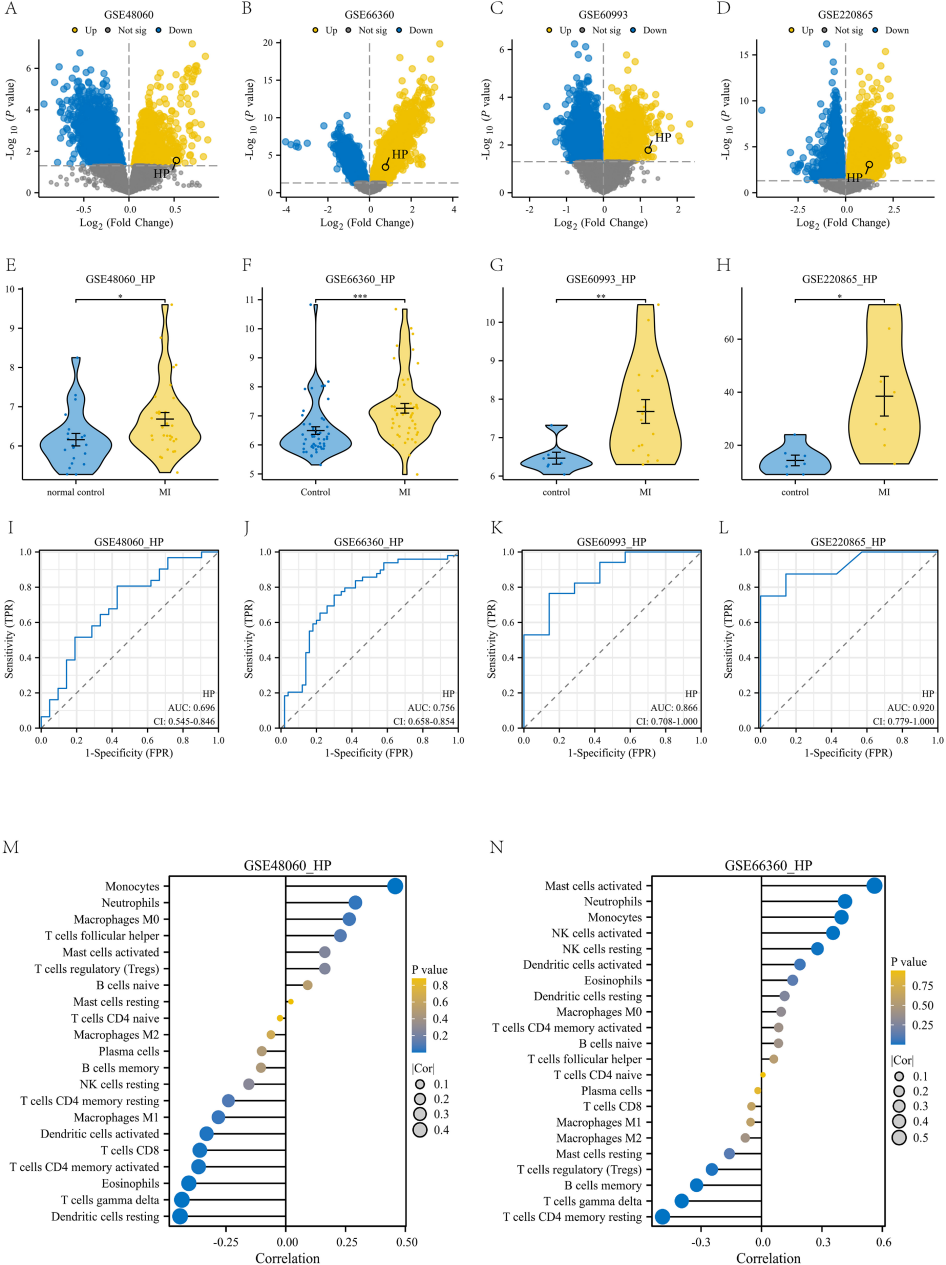
Validation with four independent datasets. **(A-D)** Volcanic maps of four independent datasets: GSE48060, GSE66360, GSE60993, and GSE220865; **(E-H)** Violin plot of differential expression of HP gene in these four independent datasets. **(I-L)** Diagnostic ROC plots of HP gene in these four independent datasets; **(M)** The correlation between HP gene and various immune cells in the GSE48060 dataset; **(N)** The correlation between HP gene and various immune cells in the GSE66360 dataset.

To meet the requirements of independent validation and further enhance the credibility of our findings, we retrieved four independent datasets from the GEO database to validate the upregulation of HP in myocardial infarction (MI) and its diagnostic potential (detailed sample information can be found in **Supplementary Table 1**). Firstly, we analyzed the differentially expressed genes in the four datasets based on the criteria of p-value<0.05 and | logFC | ≥ 0, and plotted volcano plots to mark the location of HP (**Figures 8A–D**). Subsequently, violin plots were used to demonstrate the expression of HP in four datasets, which was consistent with our previous results. The expression of HP in peripheral blood of myocardial infarction patients was significantly upregulated (p<0.05, **Figures 8E–H**). It is worth noting that receiver operating characteristic (ROC) curve analysis showed that in these four independent validation sets, HP consistently demonstrated good diagnostic performance (AUC values of 0.696, 0.756, 0.866, and 0.920, respectively, **Figures 8I–L**). In addition, through immune infiltration analysis of GSE48060 and GSE66360, it was found that there was a high positive correlation between HP and classical monocytes in these two datasets (p<0.05, **Figures 8M, N**), which is consistent with the previous findings of HP being associated with classical monocytes in single-cell analysis, further demonstrating the reliability of this result. In summary, the comprehensive evidence from these four independent cohorts, spanning the levels of transcriptomics and proteomics, provides strong multi-faceted validation for HP as a biomarker that exhibits sustained dysregulation and diagnostic value through classical monocytes.

## 4 Discussion

Myocardial infarction (MI) remains the leading cause of death worldwide, and a thorough analysis of its molecular mechanisms is crucial for improving clinical prognosis. In this study, we systematically revealed for the first time the key role of Haptoglobin (HP) in the immune microenvironment of MI. We integrated weighted gene co-expression network analysis (WGCNA) with ferroptosis and exosome phenotype gene screening strategies. Using this approach, we successfully identified the HP gene from the GSE141512 dataset. The results indicate that HP is upregulated in MI and high expression in classical monocytes, with potential value as a diagnostic biomarker for MI. In addition, functional enrichment analysis showed that this gene module is mainly involved in immune related processes, indicating that HP may contribute to the pathogenesis and progression of myocardial infarction by regulating immune responses.

Importantly, HP plays a unique role at the intersection of immune regulation and ferroptosis. Mechanistically, it prevents an increase in iron load by binding to free hemoglobin, thereby limiting iron catalyzed oxidative stress, which is the central driving factor for ferroptosis (42). In the context of myocardial infarction (MI), excessive iron deposition and lipid peroxidation cause death of myocardial cells, and upregulation of HP in classical monocytes may represent a compensatory response to alleviate the damage associated with ferroptosis (43). In addition, HP has been reported to regulate macrophage polarization and cytokine release, indicating that its overexpression in classical monocytes may directly affect the balance between pro-inflammatory and reparative immune responses after MI (44). These dual functions highlight the role of HP as a regulator of ferroptosis and immune regulation, providing a reasonable mechanistic basis for its diagnostic and therapeutic potential. Our study confirms the previous observation that HP is elevated in the plasma of patients with myocardial infarction (45). However, in addition to its known role as a plasma protein, our single-cell RNA sequencing (scRNA seq) analysis provides new insights, identifying classical monocytes as the main source of elevated HP in the MI immune microenvironment.

We validated our findings using two independent datasets (GSE141512 and GSE95368). These analyses confirmed that HP is significantly overexpressed in myocardial infarction (MI) samples and shows excellent diagnostic efficacy in proteomic data (AUC=0.833). Notably, our immune infiltration analysis using the CIBERSORT algorithm revealed a significant correlation between HP expression and immune cell subpopulations, particularly classical monocytes. This finding was further corroborated by single-cell RNA sequencing analysis of the GSE269269 dataset, which showed that HP is predominantly expressed in classical monocytes. Furthermore, we validated the significant upregulation of HP in classical monocytes from MI patients using qPCR experiments on clinical samples. This step completed the research process, bridging bioinformatics discovery and experimental verification. Importantly, the core finding of HP upregulation in MI was robustly validated across multiple independent external cohorts (GSE66360, GSE48060, GSE60993, GSE220865). This multi-cohort validation strategy directly addresses the necessity of independent replication in biomarker discovery. HP showed consistent diagnostic performance, with significant AUC values across all datasets. This consistency greatly enhances the credibility of our initial findings. It also reduces concerns about overfitting or cohort-specific biases common in studies using a single dataset. Additionally, immune infiltration and immunological correlation analyses in GSE66360 and GSE48060 further demonstrated a significant positive correlation between HP and classical monocytes, highly consistent with previous conclusions. This converging evidence elevates HP from a preliminary finding to a reliably validated candidate biomarker.

This study presents an important innovation: the precise analysis of HP’s cellular localization in the immune microenvironment of MI. This was achieved using single-cell sequencing technology. Our pseudo time trajectory analysis indicates that classical monocytes with high expression of HP mainly appear in the middle and late stages of immune response, which coincides with the tissue remodeling process after MI. This suggests that HP may be related to maintaining inflammation and coordinating repair processes, especially in the subacute stage of myocardial infarction. In addition, intercellular communication analysis showed that classical monocytes with high expression of HP actively interact with CD4+T cells and dendritic cells through cytokine and chemokine signaling pathways, which may amplify the activation of adaptive immunity. These findings suggest that HP may not only label a pathogenic subpopulation of classical monocytes, but also influence the development trajectory of post infarction inflammation by promoting interactions between classical monocytes, T cells, and dendritic cells. We speculate that the co-expression of HP in gene modules related to immune activation and ferroptosis suggests a possible link between HP and these processes; thus, the upregulation of HP in classical monocytes may represent a compensatory mechanism to counteract oxidative stress and iron overload, hallmarks of ferroptosis (46). In addition, HP is conserved in mammalian evolution and is associated with immune regulation in various species, indicating its broader role in comparative immunology (47, 48). Future cross species studies may elucidate its conserved functions in inflammatory response and tissue repair.

Although this study provides evidence supporting the role of HP in myocardial infarction, some limitations still need to be acknowledged:

1. Limited sample size: The main limitation of this study is the relatively small sample size in our clinical validation cohort (n=5 per group), which may limit the statistical power and generalizability of our research results.
2. Lack of functional experimental evidence: The functional insights we obtained mainly rely on bioinformatics inference. Although we have provided *in vitro* validation of HP expression, it is still necessary to clarify its causal relationship through functional enhancement or inhibition experiments to elucidate the specific mechanism of HP in the immune microenvironment.
3. Incomplete characterization of regulatory network: Although we constructed a preliminary ceRNA network for HP, the interactions between HP, miRNA, and lncRNA remain predictive and require experimental validation.
4. Limitations of single-cell resolution: Although we used single-cell RNA sequencing data to infer the cellular localization of HP, this lacks direct experimental evidence. Therefore, more precise experimental methods are needed to verify the expression and function of HP in different cell types.

In response to these limitations, future research will involve larger multicenter prospective cohorts to validate the diagnostic and prognostic value of HP in classical monocytes. Secondly, future research will include *in vivo* and *in vitro* functional experiments, such as knocking out or overexpressing HP in cell lines and animal models, to elucidate its role in monocyte activation and iron deposition regulation. Thirdly, future work will include using techniques such as luciferase reporter assay and RNA immunoprecipitation to validate the binding relationship between HP and identified miRNAs. Finally, spatial transcriptomics and multiplex immunofluorescence staining will be used to visualize and confirm the expression and distribution of HP in myocardial tissue slices at single-cell resolution. By addressing these limitations, future research will enhance the depth and clinical relevance of our findings, ultimately contributing to the development of HP basic diagnosis and treatment strategies for myocardial infarction.

In conclusion, this study establishes Haptoglobin (HP) as a clinically promising diagnostic biomarker and therapeutic target for myocardial infarction through integrated multi-omics analysis and experimental validation. HP demonstrates consistently high diagnostic accuracy across both transcriptomic and proteomic platforms, and its specific overexpression in classical monocytes was confirmed in clinical samples. These findings highlight the potential of HP as a practical blood-based biomarker for early MI detection and patient stratification. Further development of HP-targeted detection assays and investigation into its immunomodulatory functions could open new avenues for clinical translation. Ultimately, these advances may contribute to improved diagnosis and immune-targeted therapy for myocardial infarction.

## Availability of data and materials

The data are available on GEO database (https://www.ncbi.nlm.nih.gov/geo/).

## Data Availability

The original contributions presented in the study are included in the article/**Supplementary Material**. Further inquiries can be directed to the corresponding authors.
